# Prevalence of dizziness in the population of Minas Gerais, Brazil, and its association with demographic and socioeconomic characteristics and health status^☆^

**DOI:** 10.1016/j.bjorl.2016.01.015

**Published:** 2016-05-03

**Authors:** Tiago Ferreira Martins, Patrícia Cotta Mancini, Luiza de Marilac de Souza, Juliana Nunes Santos

**Affiliations:** a Universidade Federal de Minas Gerais (UFMG), Programa de Pós-graduação em Ciências Fonoaudiológicas, Belo Horizonte, MG, Brazil; b Fundação João Pinheiro, Belo Horizonte, MG, Brazil; c Universidade Federal dos Vales do Jequitinhonha e Mucuri (UFVJM), Teófilo Otoni, MG, Brazil

**Keywords:** Dizziness, Epidemiology, Brazilian Unified Health System, Tontura, Epidemiologia, Sistema Único de Saúde

## Abstract

**Introduction:**

The state of Minas Gerais, Brazil has no data on the prevalence of dizziness in the population and this information can be fundamental as the basis of public health policies, promotion, prevention and rehabilitation campaigns.

**Objective:**

Investigate the prevalence of the symptom of dizziness in the population of Minas Gerais according to Sample Survey of Households, as well as describe the profile of interviewed individuals and the association between dizziness and socioeconomic, demographic features and health status.

**Methods:**

This was a cross-sectional observational study that analyzed individuals with dizziness symptom reported in the previous month. The data entered in the Sample Survey of Households of 2011 were analyzed. An independent statistical association was determined between the selected variables and dizziness through multivariate analysis.

**Results:**

Dizziness was the third major complaint among individuals who mentioned any health problems in the previous month, with an estimated population of 209,025 individuals and reported by 6.7% of symptomatic ones, with higher prevalence values only reported for the symptoms of fever and headache. Among individuals who reported dizziness, 94% were adults or elderly (*p* ≤ 0.001) and 63% were females (*p* = 0.003). A statistically significant association (*p* < 0.001) was observed between the response variable and the variables: self-perceived health, hypertension, heart disease, diabetes, depression, seeking or requiring medical or health care in the previous month and private health care plan or insurance. Among individuals with dizziness, 84.2% sought or required medical or health care and 80.1% did not have a private health plan or insurance in the assessed period.

**Conclusion:**

The dizziness symptom was highly prevalent in the population of Minas Gerais during the assessed month of the investigation. Dizziness was prevalent in adults and the elderly and showed a statistical association with socioeconomic and demographic characteristics, as well as the assessed health status.

## Resumo

**Introdução:**

O estado de Minas Gerais–Brasil não possui dados sobre a prevalência de tontura na população e estas informações podem ser fundamentais para basear políticas de saúde pública, campanhas de promoção, prevenção e a reabilitação.

**Objetivo:**

Investigar a prevalência do sintoma de tontura na população do estado de Minas Gerais segundo a Pesquisa por Amostra de Domicílio (PAD-MG), assim como descrever o perfil dos indivíduos entrevistados e as relações entre tontura e características socioeconômicas, demográfica e condições de saúde.

**Método:**

Estudo de caráter observacional transversal com análise dos indivíduos com relato de sintoma de tontura no último mês. Foram analisados os dados inseridos na PAD-MG de 2011. Determinou-se associação estatística independente entre as variáveis selecionadas e a tontura por intermédio de análise multivariada.

**Resultados:**

A tontura foi à terceira queixa principal entre os indivíduos que mencionaram algum problema de saúde no último mês, com estimativa populacional de 209.025 indivíduos e relatada por 6,7% dos sintomáticos, com valores inferiores somente aos sintomas de febre e dores de cabeça, respectivamente. Dentre indivíduos com relato de tontura, 94% são adultos ou idosos (*p* ≤ 0,001) e 63% do sexo feminino (*p* = 0,003). Foi encontrada associação estatisticamente significante (*p* < 0,001) entre a variável resposta e as variáveis autopercepção de saúde, hipertensão, doenças cardíacas, diabetes, depressão, procura ou necessidade de atendimento médico ou de saúde no último mês e presença de cobertura de plano ou seguro-saúde. Dentre os indivíduos com tontura, 84,2% procurou ou precisou de atendimento médico ou de saúde e 80,1% não possuíam cobertura de plano ou seguro-saúde no período pesquisado.

**Conclusão:**

O sintoma de tontura se mostrou altamente prevalente na população de Minas Gerais no mês de referência da pesquisa. A tontura foi prevalente nos indivíduos adultos e idosos e apresentou associação estatística com as características socioeconômicas, demográficas e condições de saúde estudadas.

## Introduction

In the event of a conflict in the integration of sensory information that aids postural control, we are faced with a body balance dysfunction that can be translated as dizziness. According to the Hearing and Balance Committee of the American Academy of Otorhinolaryngology and Head and Neck Surgery, dizziness is any illusory sense of movement without real movement in relation to gravity.^1^ Dizziness may cause fear of movement, gait alterations, anxiety, insecurity, depression and fear, in addition to secondary autonomic symptoms, such as sweating, nausea and vomiting.^2^, ^3^ Dizziness often impairs social, family and work activities and causes physical, financial and psychological damage, in addition to resulting in a decrease in concentration and performance, leading to poor quality of life.^2^ This symptom is also associated with the use of five or more medications, the presence of postural hypotension and a history of acute myocardial infarction.^4^

Dizziness has been characterized as a multifactorial health condition that arises from the cumulative effect of multiple system deficits, resulting in increased vulnerability, mainly among the elderly.^4^, ^5^ There is an association between chronic dizziness and depressive symptoms, poor self-perceived health status and restricted participation in social activities.^4^, ^6^ In a two-year longitudinal study, the main factors related to dizziness in the elderly were: age, female gender, cardiovascular disease, osteoporosis, depression, sleep and memory disorders, impaired vision, incontinence, three or more comorbidities, polypharmacy, poor self-perceived health status, falls and mobility problems.^7^

Dizziness is common symptom, that is often underestimated and untreated by health professionals. It is estimated that 23.3% of patients aged 18–64 seeking a general practitioner have experienced some type of dizziness in the previous month and almost 30% of these individuals experienced dizziness in the last five years.^8^ In a study of 1000 patients with longitudinal follow-up, dizziness was the third most frequent clinical symptom in a general outpatient clinic, second only to chest pain and fatigue.^9^ The incidence of dizziness significantly increases with advancing age.^7^, ^10^, ^11^, ^12^, ^13^ In a longitudinal study of 620 elderly individuals from Germany, it was observed that the prevalence of dizziness complaint in the last 6 months was 27% among those aged up to 70 years and 54% in those aged 90 years and older.^7^ A Swedish National Study on Aging and Care with 1273 individuals observed a prevalence of dizziness in 31% of individuals aged >80 years.^14^

According to studies, the annual rates of medical consultation because of dizziness in primary care range from 2.5% in patients aged 25–44 years to 8.3% in patients aged 65 years or older, and is 18.2% in patients aged 85 years or older.^8^, ^15^ Much of the care provided to patients with dizziness is performed in primary care settings; in a study in the Netherlands carried out from 1985 to 1995, family physicians reported that only 3% of the elderly with dizziness are referred to a medical specialist.^16^

In Brazil, there are few population-based studies in the health area and very few of these investigate symptoms in the population. The PNAD (National Survey by Household Sample of the Brazilian Institute of Geography and Statistics – Instituto Brasileiro de Geografia e Estatística – IBGE) has invested in issues directed at the health status of the Brazilian population with a national scope since 2004.^17^ Updated population-based information is essential for societal planning and monitoring in various geographic and socioeconomic settings, to allow compliance with the constitutional principles of health, such as the right to equal access to and financing of health services. The dissemination of this information by IBGE also increases the possibility of incorporating health information by different areas of government, which is important to strengthen intersectoral actions, in order to follow the policies aimed to improve the overall health of the Brazilian population.^18^

The public and private health care network in the state of Minas Gerais, Brazil, does not have data on the prevalence of dizziness within the population. Thus, the analysis and dissemination of this information can help define the profile of the symptomatic population, provide additional data on determinant factors and associated comorbidities that may be crucial to establish public health policies, obtain resources, carry out promotion, prevention and rehabilitation campaigns aimed at the target population. Considering more than 170 million Brazilians do not have private health care plans or insurance and depend only on the Brazilian Unified Health System (SUS) for health care,^17^, ^19^ studies such as this investigation become essential.

Thus, the present study aims to investigate the prevalence of dizziness symptom within the population of the state of Minas Gerais, Brazil, describe the profile of the individuals interviewed by the PAD-MG who reported dizziness in the last 30 days, as well as assess associations between dizziness and demographic and socioeconomic characteristics and health status of respondents.

## Methods

This is a cross-sectional, observational study with the analysis of individuals from Minas Gerais that reported the symptom of dizziness in the last 30 days. The study comprehends the analysis of data from the PAD-MG of Fundação João Pinheiro, which is carried out similar to the National Survey by Household Samples, the IBGE-PNAD and has a sampling statistical operation.^17^

The Sample Survey of Households (PAD-MG) was developed in the state of Minas Gerais (MG); the project began in 2007 and the first research was carried out in 2009, designed to produce regionalized information able to coordinate with the monitoring and assessment of public policies. The PAD aims to obtain information on the population of different regions of MG and to be a step toward the construction within the state of an agile and flexible structure capable of meeting the specific demands of their actions. The PAD-MG is a key step in the consolidation of a state concept that accompanies the processes and the results of actions.^20^

The second round of the PAD-MG, held in 2011, was a socioeconomic survey based on a sample of 18,000 households distributed in 1200 census sectors and 428 municipalities, with regional representation for the 12 mesoregions of the state. Every two years the PAD collects information on health, education, work, income and benefits, characteristics of households and individuals and other subjects. The data helps to direct efforts and resources that are specific for the different regions of the state. The PAD-MG includes the resident population in permanent private households, but excludes residents in institutional collective household establishments.^20^

The basic registration for the selection of census sectors in PAD-MG 2011 was obtained from the Aggregated Files of the Demographic Census Synopsis Sectors of 2010 in the state of Minas Gerais, using a probabilistic sampling method. In the first stage, the initial allocation of the household and sector samples was performed using the power allocation method, while respecting the limits of 3000 households for the rural part of the sample and 15,000 homes for the urban part of the sample. In the second phase, adjustments were made to the initial allocation taking into account estimates of standard errors from the PAD-MG 2009 microdata. Moreover, an additional stratification method was applied, that consisted of organizing the sectors by microregion, municipality, district, subdistrict and neighborhood.^20^

After the organization of the census sectors, sample areas were defined, which were as many as the sample size of sectors in the stratum divided by two, aiming to select two sectors per sampling zone. The selection of two sectors per zone was carried out by Poisson sequential sampling. The total number of households in the sector was used as a measure of size. It is noteworthy that this number was truncated at 30 at the lower limit and 600 at the upper limit to reduce the variability of sector inclusion probability. For the selection of households in each sample sector of the first stage, the inverse sampling of households was proposed. This procedure allowed us to control the final size of the household sample that was actually interviewed, ensuring that the effective sample size will be equal to or very close to the size specified in the initial design.^20^

Data collection took place from 10/01/2011 to 29/02/2012, using a laptop during the face-to-face interviews. The interviewers were hired and trained by Fundação João Pinheiro for the home visits.^20^

The survey questionnaire was divided into ten sections, respectively: household characteristics; characteristics of residents; education; health, work and child labor; income; entrepreneurship; household's collective expenses (eighth and ninth); individual expenses of each resident of the household. This study will emphasize the analysis of the “health” section questions, considering as the response variable: feeling ill, with dizziness, in the last 30 days. The explanatory variables were: gender, age, seeking and requiring health care in the last 30 days, private health plan or health insurance coverage, health self-perception, follows nutritional guidelines, current smoker and the presence of health problem that requires constant monitoring.

For the description of the question “Did [Name] feel ill, with a health problem symptom in the last 30 days? (Name the main symptom in this period.)”, the respondent mentioned dizziness or one of fourteen other response options including“no symptoms”. As the study of the response variable was dizziness, the study subjects were divided into two groups, the ones who experienced dizziness during the previous 30 days and those who did not. For the description of the variable gender, the respondent answered male or female. For the description of the variable age, the respondent reported his/her age and later the interviewer classified it according to categories: adult (19–59 years) and elderly (60 years or older). For the description of the question on self-perceived health status, the respondent answered the question “How do you assess the health status of [name]?”, of which answers were provided using a five-point Likert scale (very good/good/fair/poor/very poor).

However, for data analysis, responses were grouped into good (very good and good) and poor (fair, poor and very poor). For the description of the questions “And [Name] followed these recommendations (nutritional guidelines from a doctor or nutritionist)?”, “Does [Name] smoke cigarettes now?”, “Has a doctor or health care professional said that [Name] has cardiac diseases (heart diseases)?”, “Has a doctor or health care professional said that [Name] has hypertension (high blood pressure)?”, “Has a doctor or health care professional said that [Name] has depression?”, “Has a doctor or health care professional said that [Name] has diabetes?”, “Did [Name] need or seek medical or health care in the last 30 days? “and” Does [Name] have a private health plan or insurance coverage?”, the answers of respondents were grouped into “yes” or “no” for data analysis.

The responses to the PAD-MG questions about seeking and requiring health care services considered September 1, 2011 as the reference date. The month of August and the week of August 28–31 to September 1–2 were considered, respectively, the study reference month and week.

At the PAD/MG, the free and informed consent form was replaced by verbal consent of the respondent, obtained at the time of the interview. The interviewers informed the residents about aspects of the research, its benefits, impact and importance in the evaluation of state policies and asked for their consent to participate in the research. Residents were free to accept or to refuse to participate. This study was approved by the Research Ethics Committee under ETIC protocol number 0347.0.203.000-10.

Based on the respondents’ answers, a database was generated using SPSS (Statistical Package for Social Sciences), version 19.0. Through the sampling process used in the PAD-MG, the population estimate for the state of Minas Gerais was carried out.^20^ First, a descriptive analysis of the data was performed. Then, the analysis of factors associated with dizziness was performed, with an inferential analysis using Pearson's Chi-square test for categorical variables, considering as statistical significance the 95% confidence interval (first phase). Subsequently, all the variables associated with dizziness at *p* ≤ 0.10 were tested. The variables that remained statistically associated with dizziness at *p* ≤ 0.05 remained in the final model. This analysis was performed in two stages, using binary logistic regression.

## Results

In a population estimate based on the PAD/MG 2011 sample, of a total of 19,442,971 individuals, 3,586,973 (18.44%) had a health problem symptom in the previous 30 days, with the most common symptoms being shown in Table 1.

**Table 1:** Population estimate of individuals interviewed in the PAD-MG 2011 who felt ill and had a health problem symptom over the past 30 days, mentioning only the main symptom experienced in this period.

Symptom	*n*	Relative frequency (%)	Cumulative frequency (%)
*Fever*	316,004	7.88	7.88
Diarrhea	109,900	2.87	10.75
Toothache	70,035	1.93	12.68

*Headaches*	611,080	16.13	28.81
Chest pain	99,983	3.29	32.10
Abdominal pain	178,654	4.97	37.06
Earache	29,953	0.93	37.99
Breathlessness	109,370	3.16	41.15
Bleeding	16,805	0.53	41.68

*Dizziness*	209,025	6.70	48.38
Cough	136,264	3.65	52.03
Vomiting	51,523	1.42	53.45
Other	1,648,377	46.55	100.00
Total	3,586,973	100.00	

Source: Sample Survey of Households of Minas Gerais (PAD-MG). Fundação João Pinheiro, 2011.

**Figure 1:**
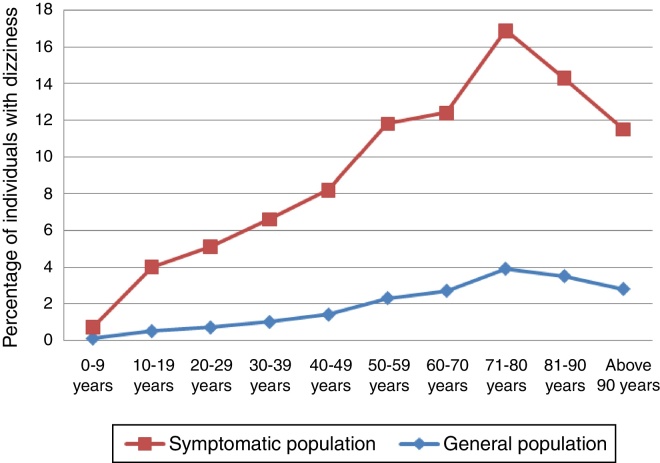
Prevalence of dizziness according to age.

*Source*: Sample Survey of Households of Minas Gerais (PAD-MG). Fundação João Pinheiro, 2011.

The mean age of the subjects with symptoms was 41.08 years and for the total sample, 35.8 years. The distribution of the stratified percentage in a 10-year scale of individuals that had dizziness in the previous month, comparing the population of individuals who reported a health problem symptom and the total sample population can be seen in Fig. 1.

In the univariate analysis, all assessed variables (gender, age, poor health self-perception, presence of chronic diseases, seeking health care services, lack of health insurance, lack of physical activity, current smoker, does not follow nutritional guidelines) showed a statistical association (*p* < 0.001) with the response variable. However, the variables gender and diabetes showed no statistical association in the multivariate analysis, even with a higher prevalence of the female gender and presence of diabetes in individuals with dizziness in relation to the total population, being respectively 131,686 (63%) and 37,209 (17.9%) among individuals with dizziness in population estimates.

The multivariate analysis of the presence of dizziness in the previous month and its association with demographic variables, health status and socioeconomic characteristics that disclosed a statistical association can be seen in Table 2.

**Table 2:** Multivariate binary logistic regression of factors associated with the presence of dizziness in the 30 days preceding the interview of PAD-MG, 2011.

Symptom of dizziness in the last 30 days						
	Yes	No		*p*-Value^b^	Odds Ratio^b^	
Variables	*n* (%)	*n* (%)	Total *n*			95%CI^b^
*Age range*						
Adults	128,237 (65.2)	11,669,927 (83.9)	11,798,164(83.7)	<0.001	1.111	1.089–1.113
Elderly^a^	68,311 (34.8)	2,239,402 (16.1)	2,307,713 (16.3)			
Total	196,548 (100)	13,909,329 (100.0)	14,105,877 (100.0)			

*Self-perception of health*						
Good	71,140 (34.0)	15,937,853 (82.9)	16,008,993 (82.4)	<0.001	1.498	1.464–1.563
Poor^a^	137,885 (66.0)	3,292,153 (17.1)	3,430,038 (17.6)			
Total	209,025 (100.0)	19,230,006 (100.0)	19,439,031 (100.0)			

*Hypertension*						
No	97,918 (46.9)	16,245,918 (84,6)	16,343,836 (84.2)	<0.001	2.000	1.965–2.053
Yes^a^	111,034 (53.1)	293,669 (15.4)	3,064,703 (15.8)			
Total	208,952 (100.0)	19,199,587 (100.0)	19,408,539 (100.0)			

*Cardiac diseases*						
No	166,636 (79.9)	18,340,070 (95.5)	18,506,706 (95.4)	<0.001	1.166	1.141–1.191
Yes^a^	42,004 (20.1)	860,293 (4.5)	902,297 (4.6)			
Total	208,640 (100.0)	19,200,363 (100.0)	19,409,003 (100.0)			

*Depression*						
No	156,072 (74.8)	18,389,068 (95.8)	18,545,140 (95.5)	<0.001	1.963	1.923–2.005
Yes^a^	52,687 (25.2)	812,086 (4.2)	864,773 (4.5)			
Total	208,759 (100.0)	19,201,154 (100.0)	19,409,913 (100.0)			

*Current smoker*						
No	168,817 (83.4)	13,189,748 (86.9)	13,358,565 (86.9)	<0.001	1.134	1.105–1.164
Yes^a^	33,669 (16.6)	1,988,328 (13.1)	2,021,997 (13.1)			
Total	202,486 (100)	15,178,076 (100)	15,380,562 (100)			

*Follows nutritional guidance*						
Yes	15,499 (30.0)	699,773 (37.2)	715,272 (37.0)	<0.001	1.416	1.388–1.444
No^a^	36,215 (70.0)	1,180,612 (62.8)	1,216,827 (63.0)			
Total	51,714 (100)	1,880,385 (100)	1,932,099 (100)			

*Sought or required medical or health care in the previous month*^c^						
No	33,115 (15.8)	15,805,500 (82.2)	15,838,615 (81.5)	<0.001	8.900	8.677–9.129
Yes^a^	175,910 (84.2)	3,419,065 (17,8)	3,594,975 (18.5)			
Total	209,025 (100)	19,224,565 (100)	19,433,590 (100)			

*Has private health plan or insurance*						
Yes	41,613 (19.9)	4,065,960 (21.2)	4,107,573 (21.1)	<0.001	1.069	1.048–1.091
No^a^	167,412 (80.1)	15,150,814 (78.8)	15,318,226 (78.9)			
Total	209,025 (100)	19,216,774 (100)	19,425,799 (100)			

a Categories of reference.

b Results obtained after multivariate analysis; the final model includes the main dependent variable adjusted for other variables that remained in the final model.

c Medical or health care. # Number of information differs from the total sample due to missing data.

## Discussion

The interviewed population is representative of the state of Minas Gerais and the dizziness symptom was the third most prevalent main complaint among individuals who mentioned some type of health problem in the previous month, reported by 6.7% of symptomatic individuals, with lower values only when compared to the symptoms of fever and headache, respectively. It is estimated that of the population of 19,442,871 individuals; 209,025 experienced dizziness as the most prevalent symptom in the assessed month. According to Kroenke et al.,^9^ dizziness is the third most common clinical symptom in a general outpatient clinic. Authors of international population studies indicate that dizziness prevalence range from 11% to 32.5%.^8^, ^15^, ^21^, ^22^ Bittar et al.^23^ established a prevalence of dizziness as 42% in study carried out in São Paulo, Brazil, a higher proportion than that found in other studies. However, in a study of 4869 individuals, the prevalence of dizziness of vestibular origin in adults was estimated at 7.4%.^11^ This variation in prevalence can be influenced by methodological biases, including how the data was collected, symptom description, and mainly the prevalence measure used; some studies used the prevalence throughout life, with consequent higher values, whereas the present study used the prevalence only during the study period. Individuals interviewed in the PAD-MG only answered about the main health problem in the previous month and might also have felt dizziness as a secondary symptom and thus, they did not mention it in the survey.

The present study found that 94% of patients with reported dizziness are adult or elderly individuals, representing 196,548 individuals. Of these, the elderly have a 1.111-fold higher chance of having dizziness as the main health problem than adults, with a statistically significant association (*p* < 0.001) between the response variable and the age variable. The study observed that the prevalence of dizziness increases in direct proportion with age, with a peak between 71 and 80 years, corroborating the findings of Charles et al.^10^ and Neuhauser et al.,^11^ who reported a peak between 65 and 75 years. Moraes et al.^24^ found a prevalence of dizziness of 45% in a study with 391 elderly individuals and Olsson Möller et al.^14^ found a prevalence of dizziness of 17.8% and 31% in individuals aged <80 and >80 years, respectively. The findings of higher prevalence of dizziness in the elderly agree with several worldwide literature reports^10^, ^15^, ^21^, ^22^, ^23^, ^25^, ^26^, ^27^ and can be explained by the aging process of the balance system, multiple sensory deficits, which are common in elderly patients and accumulation of comorbidities, such as cardiovascular, metabolic and neurological diseases. In a study performed at the University Hospital of Zurich, Switzerland, with 266 individuals with dizziness older than 65 years, 37.6% were diagnosed with multisensory dizziness,^5^ which reinforces the impact of aging on the increasing prevalence of this symptom.

Among the individuals who reported dizziness, 63% were females, representing 131,686 individuals in the state of Minas Gerais and this distribution is in agreement with the literature.^25^, ^26^ The prevalence of dizziness in females has been reported in several other studies,^8^, ^10^, ^11^, ^12^, ^22^, ^23^, ^24^, ^28^, ^29^, ^30^, ^31^ which can be explained by the hormonal variations responsible for ovarian cycles and menopause,^32^, ^33^ higher prevalence of migraine,^12^, ^29^ the fact that women more often seek medical care^23^, ^28^ and a higher prevalence of women in the elderly population worldwide. However, as in this study, some researchers also found no statistical association between dizziness and gender,^15^, ^27^ as shown in the English study of 2925 individuals older than 65 years.^21^

When analyzing the association between the health status of individuals with the response variable, a statistically significant association was observed (*p* < 0.001) in the multivariate analysis with the variables self-perceived health, hypertension, heart disease and depression. It is observed that 66% of individuals who reported dizziness in the previous month showed poor self-perceived health, representing 137,885 individuals, well above the 17.1% of individuals that had other health problems symptoms, indicating a significant negative impact of dizziness on patient quality of life.

It was also verified that individuals with poor self-perceived health had a 1.498-fold higher chance of having dizziness as the main health problem than those who reported good self-perceived health. The poor perception of health associated with the complaint of dizziness corroborates other studies.^7^, ^24^, ^34^ As for individuals with hypertension, heart disease and depression they had, respectively, a 100%, 16.6% and 96.3% higher chance to have dizziness as the main health problem when compared to those who did not have this symptom. In a study of 493 elderly individuals, Lopes et al.^28^ found a statistical association between dizziness and hypertension. Dros et al.^30^ studied 417 elderly patients with dizziness in primary care in the Netherlands and found that 49% had heart disease and 57% had hypertension. The findings agree with other studies,^32^, ^35^, ^36^ which reported that dizziness can be a secondary effect of arterial hypertension and heart disease. Studies with the elderly found an association between dizziness and a positive score for depressive symptoms.^6^, ^13^, ^30^ In the 7-year follow-up of a prospective cohort with 681 elderly, Maarsingh et al.^31^ found anxiety or depression in 33.6% of patients with dizziness and in only 15.1% of individuals without dizziness, which showed a statistically significant association. According to Ekwall et al.,^13^ there is evidence that neurotological disorders are related to anxiety and increase in psychological problems, which, in turn, can aggravate the intensity of the dizziness complaint. In a study by Neuhauser et al.,^11^ depression and several cardiovascular diseases were associated with vestibular vertigo.

Although this study demonstrates that the health status is multifactorial and a result of the cumulative effects of multiple system deficits, making individuals more vulnerable during the aging process and subject to inadequate changes in balance physiology, there was no statistically significant association in the multivariate analysis between the response variable and the presence of diabetes, which corroborates other studies.^30^, ^36^ In a Brazilian study with 391 individuals older than 65 years, Moraes et al.^24^ found no statistical association of dizziness with diabetes and obesity. This study found a statistically significant association (*p* < 0.001) between the response variable and the variable current smoker, in which 16.6% of subjects with dizziness reported being smokers and they showed a 13.4% higher chance to have dizziness as the main health problem when compared to individuals who did not smoke. This finding agrees with a community-based study carried out in a university outpatient clinic.^36^ In the study by Cruz et al.^37^ with 751 young adults and using multivariate analysis, an association was found between smoking and dynamic balance test alterations. Pereira et al.^38^ suggests that nicotine can induce imbalance in the vestibular-ocular and vestibular-spinal reflexes; however epidemiological investigations of this association are still very incipient. Although a Brazilian study did not find an association between dizziness and smoking,^24^ it is known that smoking is associated with increased risk of chronic non-communicable diseases such as cardiovascular and pulmonary diseases and cancer, with a consequent impact on the physiology of the vestibular system.

When assessing the association between the variable patient follows nutritional guidelines with the response variable, a statistically significant association was observed (*p* < 0.001) and individuals who did not follow nutritional guidelines had a 1.416-fold higher chance of having dizziness as the main health problem when compared to those who followed the guidelines. No studies were found with statistical associations between following nutritional guidelines and symptoms of dizziness, but some authors report the importance of nutritional counseling in the treatment in patients with dizziness to avoid or change poor eating habits,^39^, ^40^ which facilitates better balance, cardiovascular health and metabolic disease control.

The association between the variables sought or needed medical or health care in the previous month and had health care plan or insurance coverage with the response variable showed a statistically significant association (*p* < 0.001). It was observed that among individuals with dizziness, 84.2% sought or needed medical or health care, a much higher number than the 17.8% of individuals that had another health problem symptom and sought or needed health care service. These represent 175,910 individuals with dizziness in MG and they have an 8.9-fold higher chance of having dizziness as the main health problem when compared to individuals who did not seek care.

In a study by Bittar et al.,^23^ 54% of symptomatic patients did not seek medical attention, even after dizziness started affecting the quality of life. However, in a study in Germany, 80% of patients with dizziness underwent medical examination, interruption of daily activities or went on sick leave.^11^ This difference can be explained by cultural values and easy accessibility to health care in different countries.

Regarding the socioeconomic variable, it was observed that 80.1% of patients who had dizziness did not have a private health care plan or health insurance, which represents 167,412 individuals treated by SUS in the event of demand for services during that period and the consequent impact of public spending on health. These data corroborate the information from June/2014 of the Supplementary National Health Agency,^19^ that only 26.1% of the Brazilian population has private health plan/insurance. Thus, it can be observed that SUS remains the main provider of health services used by the Brazilian population and that dizziness has a consequent high impact on the health system demand due to its prevalence.

Among the study limitations, it can be observed that the research subjects only responded concerning the main health problem in the previous month. Other subjects may have experienced dizziness as a secondary symptom and for this reason they might not have mentioned it during the investigation and they also might have experienced dizziness in other periods prior to the study reference month; in both cases this would result, in n increased prevalence of dizziness in the investigation.

## Conclusion

The dizziness symptom as the main health problem in the symptomatic population was shown to be highly prevalent and affects 6.7% of the population of Minas Gerais, which is estimated at more than 209,000 individuals with dizziness symptom during the reference month of the investigation.

Among individuals with dizziness, 94% are adults or elderly, with the elderly showing an 11.1% higher chance of having dizziness as the main health problem than adults. The incidence of dizziness increases in direct proportion to age, with a peak prevalence between 71 and 80 years and a significant increase after 50 years of age. Among individuals with dizziness, 66% reported poor self-perceived health and these had a 1.498-fold higher chance of having dizziness as the main health problem than those who reported good self-perceived health. There was a statistically significant association of dizziness with the variables blood pressure, heart disease, depression and being a current smoker in the multivariate analysis and these individuals were more likely to have dizziness as the main health problem. Individuals who did not follow nutritional guidelines have a 49.8% higher chance of having dizziness as the main health problem in relation to those who follow the guidelines.

Of the individuals with dizziness, 84.2% sought or required medical or health care, representing 175,910 individuals in Minas Gerais. It is also estimated that of those symptomatic individuals with dizziness, 80.1% or 160,412 individuals had no private health care plan or insurance coverage in the assessed period.

Therefore, we observe a great impact of dizziness in SUS and demonstrated the importance of health promotion projects and actions, aimed at dizziness prevention and intervention in the vulnerable population.

## Conflicts of interest

The authors declare no conflicts of interest.
